# Targeted Radionuclide Therapy: New Advances for Improvement of Patient Management and Response

**DOI:** 10.3390/cancers11020268

**Published:** 2019-02-25

**Authors:** Javian Malcolm, Nadia Falzone, Boon Q. Lee, Katherine A. Vallis

**Affiliations:** CRUK/MRC Oxford Institute for Radiation Oncology, University of Oxford, Oxford OX37DQ, UK; javian.malcolm@oncology.ox.ac.uk (J.M.); nadia.falzone@oncology.ox.ac.uk (N.F.); leebq0718@gmail.com (B.Q.L.)

**Keywords:** patient management, precision medicine, targeted radionuclide therapy, prostate cancer, neuroendocrine cancer

## Abstract

Compared to external beam radiotherapy, targeted radionuclide therapy (TRT) allows for systemic radiation treatment of metastatic lesions. Published work on recent strategies to improve patient management and response to TRT through individualising patient treatment, modifying treatment pharmacokinetics and increasing anticancer potency are discussed in this review, with a special focus on the application of clinically evaluated radiolabelled ligands and peptides in the treatment of neuroendocrine and prostate cancers.

## 1. Introduction

There is an unmet need to treat minimal residual disease and micrometastatic spread of tumour cells, as the current cancer treatment options like surgery and external beam radiotherapy are less effective once a tumour has disseminated. Targeted radionuclide therapy (TRT) involves the use of a radio-labelled biologic or other vehicle to target and deliver a cytotoxic amount of radiation to inoperable or disseminated disease by emitting Auger, β- or α-particles. In the past five years, three radiopharmaceuticals have been approved by the FDA for clinical use of TRT: Radium-223 (^223^Ra)-dichloride (Xofigo^®^) for the treatment of metastatic castration-resistant prostate cancer (mCRPC patients) with symptomatic bone metastases without visceral metastases, Lutetium-177 (^177^Lu)-DOTA-TATE (LUTATHERA^®^) for the treatment of somatostatin receptor-positive (SSTR) gastroenteropancreatic neuroendocrine tumours (NETs) and Iodine-131 (^131^I)-iobguane (AZEDRA^®^) for the treatment of patients with metastatic pheochromocytoma or paraganglioma. Although today, uncomplicated local or locoregional tumour control can be achieved in many patients, distant relapse rates are still substantial among some groups of patients, opening research avenues for TRT developments. 

This Review discusses three main developments for widening the therapeutic index of TRT: Improvement in selection of patient groups who are likely to experience maximum benefit with minimal normal tissue toxicity, modification of radiopharmaceutical pharmacokinetics (PK) to increase tumour uptake and decrease normal tissue uptake and improvements in the anticancer potency of radiopharmaceuticals. These developments will be discussed with a special emphasis on metastatic castration-resistant prostate (mCRPC) and neuroendocrine (NET) cancers.

## 2. Developments in Individualisation of Treatment 

As TRT becomes more available, clinically heterogeneous patient groups are being treated and there is increasing value in the identification of imaging or metabolic markers to predict tumour response as well as normal tissue toxicity in patients receiving TRT. Treatment individualisation plays a key role not only in the initial planning of TRT but in subsequent re-treatments; here dosimetry should be considered with the potential to treat with tumourcidal doses without affecting organs at risk (OAR).

### 2.1. Pre-Treatment Imaging Parameters

The utility of an imaging agent is not only the ability to evaluate treatment response, but to change treatment strategy based on the appropriate selection of responders/non-responders. Pre-treatment PET imaging has served as a primary research focus to identify patients who would benefit from or fail therapies with various radionuclide and targeting moiety combinations. For example, Vija et al. demonstrated that quantification of bone tumoural burden using 18F-Fluoromethylcholine (^18^F-FCH) PET/CT could predict normal tissue toxicity, specifically grade 2–3 haematological toxicity after ^223^Ra therapy [1]. For tumour response, Murray et al. showed that if the response is defined as a change of >21% in the mean standard uptake value (SUV_mean_) of bone lesions, the percentage change of ^18^F-fluoride PET SUV_mean_ relative to baseline could identify non-responding bone lesions prior to ^223^Ra therapy (r^2^ = 0.77) [2]. 

The level of uptake in tumour on the baseline, the PET scan has been explored as a predictor of response in SSTR-positive NETs with inconclusive evidence. ^68^Ga-DOTATOC measured as standard uptake value (SUV) on baseline PET/CT was correlated with time to progression, however this relationship could not be confirmed in the patient cohort of Soydal et al. [3]. As an alternative, the expression of SSTR quantified by SSTR-PET has been explored as a potential predictor of response to ^177^Lu-DOTA-TATE. Werner et al. [4] determined baseline intratumoural SSTR heterogeneity as assessed by SSTR-PET (^68^Ga-DOTATOC) and concluded that the four heterogeneity parameters of Entropy, Correlation, Short Zone Emphasis and Homogeneity outperform conventional PET parameters, such as mean and maximum standardized uptake value SUV mean/max in distinguishing between responders from non-responders in a retrospective cohort of 142 patients. Werner et al. further showed that an intratumoural textural feature (TF) analysis of a baseline SSTR-PET can differentiate high-risk from low-risk groups of patients with pancreatic NET tumours (pNET) scheduled for peptide receptor radionuclide therapy (PRRT) [5]. Aside from ^68^Ga-PET, SPECT scans provide an alternative imaging option. Wetz et al. showed that an ^111^In-DTPA0 octreotide SPECT scan of the spatial SSRT distribution in NET metastases as quantified by asphericity was predictive of response to PRRT [6]. 

In contradistinction to pre-treatment ^68^Ga-DOTA-peptide imaging of NET, where the uptake level in the tumour correlates with tumour differentiation, the uptake level in prostate cancer on pre-treatment ^68^Ga-PSMA PET/CT reflects the expression of prostate specific membrane antigen (PSMA), which increases as the tumour becomes metastatic and more aggressive. Limited data in the literature have shown prostate cancers with low uptake on ^68^Ga-PSMA PET/CT responds poorly to treatment [7].

### 2.2. Patient Clinical Parameters

Patient clinical parameters have also been assessed as predictive biomarkers for the TRT response. Ferdinandus et al. determined that low platelet counts and permanent use of an analgesic medication for bone pain are negative predictors of therapy response to ^177^Lu-PSMA-617 among mCRPC patients [8]. In addition to this response, a study by Ahmadzadehar et al. showed prostate-specific antigen (PSA) level was highly predictive of overall survival (OS) following the first cycle [9]. Compared to other clinical parameters of alkaline phosphatase, lactate dehydrogenase and C-reactive protein, a decline in PSA levels of more than 14%, measured two months after the first cycle of TRT was the most important response parameter with regard to OS. 

Indices composed of a combination of clinical parameters have been explored as predictive of response to TRT for NET. The ‘PRRT predictive quotient’ (PPQ) was introduced by Bodei et al. as an attempt to use whole blood gene-expression profiling, tumour grade and circulating Chromogranin A (CgA) levels to predict PRRT responders and non-responders [10]. In a cohort of 72 patients, the PPQ displayed an effectiveness of 95% with responders correctly predicted in 94–97% and non-responders in 93–100% of cases. In an attempt to address the technical and financial barriers to PPQ, Black et al. introduced the inflammation-based index (IBI), derived from the serum C-reactive protein and albumin levels, both markers of systemic inflammation, to predict progression free survival (PFS) and OS of NET patients treated with ^177^Lu-DOTA-TATE [11]. Another patient characteristic-based index was reported by Weiner et al. who identified that clinical parameters of lower aspartate transaminase (AST; ≤40 IU/L), lower carcinoembryonic antigen (CEA; ≤20 ng/mL), lower neutrophil-lymphocyte ratio (NLR; <5), and absence of the extrahepatic tumour burden were independently associated with higher OS after yttirum-90 (^90^Y) hepatic radioembolization in colorectal cancer patients [12].

### 2.3. Patient-Individualised Dosimetry

There are currently no universally accepted standardized protocols for tumour dosimetry, however several centres have successfully integrated dosimetry as part of TRT [13,14]. For a summary of the main dosimetry methodologies for TRT in NET, their drawbacks and appropriate use followed by a structured overview of clinical applications readers are directed to these recent review articles [15,16]. 

Individualised dosimetry using patient-based radiopharmaceutical biodistribution, tissue densities and anatomical geometry may allow a safe increase of administered activity of ^177^Lu-DOTA-TATE in NET patients.

Del Prete et al. compared an empiric, fixed activity treatment protocol (7.4 GBq per cycle) to a personalised patient-specific treatment protocol during a four-cycle course in a retrospective study of 36 NET patients [17]. He found the personalised protocol resulted in an average 1.48-fold increase in cumulative maximum tumour absorbed dose compared to empiric PRRT. In a larger study of 200 NET patients, Garske-Román et al. highlighted the importance of individualised kidney dosimetry in planning ^177^Lu-DOTA-TATE treatments [18]. Patients in whom the absorbed dose to the kidneys reached 23 Gy had a longer OS than those in whom it did not. Dosimetric analysis has also been shown to be feasible for concurrent treatment of ^177^Lu-DOTA-TATE plus radio-sensitising chemotherapy (capecitabine and temozolomide) [19]. While quantitative SPECT/CT techniques can provide verification of radiation dose delivered from each cycle of PRRT, PET/CT-based dosimetry has the potential for greater quantitative capability for individualised prospective dosimetry due to its comparatively higher resolution. However, the short half-life of ^68^Ga-DOTA-TATE is too short to model the retention and clearance kinetics required for accurate dosimetry estimation in individual patients. In a first-in-human prospective trial of 10 NET patients, Hicks et al. showed the utility of ^64^Cu-SARTATE as a suitable PET tracer for multiple time-point prospective dosimetry estimation with the quality of images obtained twenty-four hours following ^64^Cu-SARTATE being superior to those obtained at 1 hour with ^68^Ga-DOTA-TATE [20].

## 3. Developments in Modifying Pharmacokinetics

These advancements have focused on increasing tumour uptake and at the same time decreasing uptake in normal healthy tissues that express physiologic levels of the target, and on reducing concentration of the therapeutic radiopharmaceutical in excretory organs such as the kidney.

### 3.1. Albumin Binding

Human serum albumin targeting is ideal for compartmentalising radiopharmaceuticals because the protein is abundant in blood serum, has a long physiologic half-life, and is known to reversibly bind small negatively charged or hydrophobic molecules. This strategy may enable less frequent administration and a reduction in the amount of administered radioactivity compared to the current vehicles. Several pre-clinical studies have shown significantly higher uptake and retention with novel ^177^Lu and ^90^Y-labelled constructs for albumin binding using an Evans blue (EB) derivative for albumin [21,22,23]. Kuo et al. designed and evaluated a novel albumin-binder-conjugated to PSMA-617 derivative labelled with ^177^Lu, ^177^Lu-HTK01169 [24]. This was shown in a murine model (LNCaP tumour xenografts expressing PSMA) to have an extended blood retention time, with delivery of an 8.3-fold higher absorbed radiation dose compared to ^177^Lu-PSMA-617. In a first-in-human study with eight patients, Zhang et al. showed that ^177^Lu-DOTA-EB-TATE extended circulation in the blood and achieved a 7.9-fold increase in tumour dose delivery compared to ^177^Lu-DOTA-TATE with no reported adverse symptoms [25]. However, with the enhanced tumour dose from increased circulation time, there was an 18.2-fold increase in red-marrow dose; which decreases the ratio of tumour to dose-limiting organ dose. 

A potential drawback to the use of low-molecular-weight small-molecule PSMA inhibitors in the treatment of prostate cancer, relates to their rapid redistribution in the body and localization to the parotid, salivary, and lacrimal glands as well as to the kidney, leading to dose-limiting toxicities. To facilitate prolonged circulation time and greater tumour targeting, a dual-target binding approach, ^131^I-RPS-027, using a urea-based ligand with high affinity for PSMA and human serum albumin (HAS) has been proposed [26]. In a preclinical model of prostate cancer ^131^I-RPS-027 demonstrated a higher therapeutic index relative to MIP-1095 (a PSMA ligand in current clinical use). ^131^I was replaced by the therapeutic radiohalogen ^211^At, allowing this ligand to be considered for treatment of prostate cancer by targeted α-therapy.

### 3.2. Antagonists

Somatostatin receptors have been successfully used as targets for both imaging and therapy in patients with NET neoplasia’s for over 20 years [27]. A relatively new development is the use of SSTR antagonists, which seem to confer greater specificity, more favourable pharmacokinetics and better tumour visualization than agonists [28]. Recently, two novel agents have been evaluated in the clinical setting: ^68^Ga-OPS202 (^68^Ga-NODAGA-JR11) and ^68^Ga-DOTA-JR11 are companion PET imaging agents to a therapeutic counterpart, ^177^Lu-OPS201 (^177^Lu-DOTA-JR11). 

The superiority of the SSTR antagonist, ^68^Ga-OPS202, over the agonist, ^68^Ga-DOTATOC, was first demonstrated in a phase I study and recently confirmed in a prospective phase I/II study [29,30]. Not only did the antagonist have significantly higher lesion-based overall sensitivity than the agonist-based scans: 94% and 88% for 50 µg and 15 µg ^68^Ga-OPS202 and 59% for 15 µg ^68^Ga-DOTATOC, respectively (*p* < 0.001), but there were also no significant differences in image contrast, sensitivity, or positive predictive values between the two ^68^Ga-OPS202 peptide doses used. The corresponding therapeutic agent, ^177^Lu-OPS201, was associated with tumour doses that were 1.7- to 10.6-fold higher than those observed with the SSTR agonist ^177^Lu-DOTA-TATE in four patients with metastatic NET [31]. Furthermore, ^177^Lu-OPS201 exhibited higher tumour uptake, longer tumour residence time, and improved tumour-to-kidney dose ratio compared to ^177^Lu-DOTA-TATE and ^90^Y-OPS201 [32]. These findings gave rise to an international multi-centre, open-label study opened in March 2017 with an estimated completion date of May 2022, to evaluate the safety, tolerability, biodistribution, dosimetry and preliminary efficacy of ^177^Lu-OPS201 (ClinicalTrials.gov identifier: NCT02592707) in patients with SSTR-positive NET. In addition, the evaluation of the theranostic pair ^68^Ga-OPS202 and ^177^Lu-OPS201 in patients with SSTR-positive NETs, is currently ongoing in a single centre study (ClinicalTrials.gov identifier: NCT02609737).

In a prospective study, 20 patients with advanced NET were evaluated using another antagonist PET imaging tracer, ^68^Ga-DOTA-JR11 [33]. As with ^68^Ga-OPS202, ^68^Ga-DOTA-JR11 showed rapid tumour uptake, high tumour/background ratios and rapid blood clearance. Interestingly, little or no uptake above background was seen in the pituitary gland, spleen, adrenals and uninvolved liver compared to the known biodistribution of somatostatin receptor agonists. This pattern was also observed with ^68^Ga-OPS202 and confirms the potential to improve current imaging and therapy practices for NET. Due to the superior affinity of SSTR antagonists for the receptor compared to agonists, an important aspect that warrants further investigation is the extension of this approach to tumour types with lower SSTR expression that are not currently investigated or treated with SSTR-targeted agents, such as breast, small cell lung, renal and medullary thyroid cancer, non-Hodgkin lymphomas, pheochromocytomas and lung NETs [28]. 

An interesting new theranostic option for gastrin-releasing peptide receptor (GRPR) positive cancers, is ^68^Ga- or ^177^Lu-labelled NeoBOMB1, a DOTA coupled GRPR antagonist with high GRPR affinity and in vivo stability [34]. GRPR, also known as bombesin receptor subtype 2, is a G-protein-coupled receptor mostly expressed in organs of the gastrointestinal tract and the pancreas but also in various cancers including breast and prostate cancer. Dalm et al. reported excellent tumour uptake and favourable pharmacokinetics in a human prostate cancer xenograft model in mice [34]. This highlights GRPR as an interesting target for radionuclide therapy of prostate cancer, especially with regards to its low expression in the salivary glands, unlike PSMA, which is known to result in xerostomia. However, one downside of this target is its high expression in low grade prostate cancer compared with high grade tumours, which are a greater challenge for treatment. Never the less, this radiotracer holds promise for imaging and therapy of GRPR-expressing tumours. 

### 3.3. Radioprotectors

Protection of normal organs through reduction of off-target uptake allows greater amounts of radioactivity to be delivered, which may increase the efficacy of treatment. For TRT, the kidneys represent a major organ at risk. Kristiannson et al. showed in BALB/c mice that a radical scavenger and antioxidant, human protein α1-microglobulin (A1M), may be used as a radioprotector for kidneys during clinical PRRT with ^177^Lu-DOTA-TATE, potentially improving tumour control by allowing higher treatment activities, an increased number of fractions and obviating the need for amino acid infusions [35]. Therapy with radionuclides that emit high linear energy transfer (LET) particles, such as alpha emitters, while increasing the potency of tumour radiation, also exposes normal organs like the kidney and liver to a potentially higher dose of radiation. In a pre-clinical study, Chan et al. demonstrated renal protection in rats bearing AR42J (pancreatic) tumours, as measured by neutrophil gelatinase-associated lipocalin (NGAL) levels, when L-lysine was administered immediately prior to ^213^Bi-DOTA-TATE. In a dose escalation study L-Lysine-treated rats experienced prolonged survival compared to those without pre-administration of L-lysine, providing substantial evidence for pharmacological protection to mitigate nephrotoxicity [36]. 

Salivary gland toxicity is the most common side effect of PSMA-targeted radionuclide therapy, particularly ^225^Ac-PSMA therapy. Preventative approaches to mitigate this side effect have included intraglandular injection of botulinum toxin [37] and monosodium glutamate [38].

## 4. Developments in Increasing Anticancer Potency

Recent developments to increase the lethality of a radiopharmaceutical once it has been delivered to the tumour have been centred on the use of high LET radiation and combinations with other forms of radiation and pharmaceutical agents. 

### 4.1. High LET Radiation

β-emitting radionuclides dominate the scene as far as the targeting of solid tumours in TRT is concerned. However, targeting small metastatic disease presents a challenge as the long physical range (0.2–12 mm) of β-particles results in energy deposition beyond the boundary of small metastases. Systemic targeting with α-particle emitting radionuclides circumvents this range limitation problem, as energy deposition occurs over the same scale range as the dimension of the metastases (40–100 µm). In addition, α-particles confer a greater biological effect, due to the higher energy and LET of α-emissions (50–230 keV/µm) compared to β-particles (0.2 keV/µm). 

In recent years, targeting prostate cancer metastasis with high LET α-particles has seen success, with the first integration of an α-emitter, ^223^RaCl_2_, into treatment algorithms for patients with mCRPC following publication of the results of the ALSYMPCA trial [39]. Where mCRPC was previously associated with a very poor prognosis, there is now a drive to use α-therapy not only at an earlier stage of the disease but with curative intent [40]. High response rates with limited toxicity have been reported by several authors using the α-emitter, ^225^Ac-PSMA [41,42]. PSMA-directed α-therapy in conjunction with synergistic pharmacologic approaches such as inhibitors of DNA double-strand break repair or inhibitors of the androgen receptor may enhance radiation delivery by increasing PSMA expression in tumour cells and potentiating treatment. 

The first-in-human experience of ^213^Bi-DOTATOC in the treatment of multi-resistant NET tumours that were refractory to ^90^Y-/^177^Lu-DOTATOC showed enduring responses with favourable acute and mid-term toxicity at therapeutic effective doses [43]. This study provided clinical justification for the use of TAT in patients refractory to β-therapy and paved the way for subsequent studies with ^225^Ac-DOTATOC using dose escalation to define the maximum tolerated dose (MDT) for single cycle and fractionated delivery [44]. Another α-emitting somatostatin analogue, ^212^Pb-DOTAMTATE (AlphaMedix™), is currently being evaluated in subjects with unresectable, metastatic SSTR-positive NETs. A phase 1 trial launched earlier this year (ClinicalTrials.gov NCT number: NCT03466216) aims to assess the safety and dose limiting toxicity using ascending doses of AlphaMedix^TM^ and to assess the pharmacokinetic properties and preliminary effectiveness of the construct.

It is generally acknowledged that mAbs are suboptimal vehicles for α-emitting radionuclides due to a long serum half-life resulting in higher bone marrow toxicity and liver accumulation [45]. Advances in antibody engineering have led to antibody derivatives that are more rapidly cleared from the circulation. In particular, nanobodies—antigen-binding fragments from antibodies of Camelidae, exhibit high affinity and specificity, fast diffusion and clearance kinetics in vivo, high tumour-to-normal-tissue ratios, and good serum stability [46]. The utility of nanobodies has been demonstrated in a recent first-in-human PET study with a GMP-grade ^68^Ga-labeled anti-HER2 nanobody in HER2 positive breast carcinoma [47]. This study confirmed the fast clearance of nanobodies in patients, with only 10% of the injected activity remaining in the blood at 1 h p.i. The same group have now developed an ^225^Ac conjugated version for therapy and preliminary in vitro results show that ^225^Ac-DOTA-Nb is a promising new radio-conjugate for TAT [48]. 

### 4.2. Combinations with Other Pharmaceuticals 

There is a distinct advantage in combining systemically delivered targeted radionuclides and synergistic pharmacologic therapies in an adjuvant setting [49]. This was first proven in the treatment of thyroid cancer, where adjuvant radionuclide therapy in combination with hormone therapy has shown the potential to cure micrometastatic disease [50,51]. This same approach was taken to improve the therapeutic ratio of ^223^Ra in the treatment of mCRPC. The ERA-224 trial initiated in 2016 evaluated the efficacy of concurrent administration of ^223^Ra dichloride and the antiandrogen, abiraterone acetate, in men with castration-resistant prostate cancer (CRPC) with symptomatic bone metastases (ClinicalTrials.gov identifier: NCT03325127). This trial was the precursor to the design of the adjuvant treatment of Prostate Cancer—ADRAD trial (ClinicalTrials.gov identifier: NCT00653848), where Docetaxel is given prior to ^223^Ra. Higher PSMA expression has been seen in tumour cells exposed to anti-hormonal treatment [52,53]. Recently Luckerath and co-workers evaluated this phenomenon in a murine mCRPC model [54]. An androgen receptor blockade (ARB)-mediated increase in PSMA expression was observed with ^68^Ga-PSMA11 PET imaging and flow cytometry. However, although pre-treatment with ARB significantly increased DNA damage, it did not translate to a synergistic effect when combined with ^177^Lu-PSMA617. 

Although ^177^Lu-DOTA-TATE is one of the most promising targeted therapeutic options in NET patients, it rarely achieves a cure. Thus, different approaches are being tested to increase efficacy. The recent randomized phase III (NETTER-1) trial, showed a clear clinical advantage for the combination of PRRT, ^177^Lu-DOTA-TATE with Octreotide over Octreotide monotherapy for treating patients with unresectable GEP-NETs [27]. However, it is unclear whether the combination of PRRT and somatostatin analogues (SSA) such as Octreotide and Lanreotide provides a survival benefit when compared with PRRT alone. In a retrospective study by Yordanova et al. patients with GEP-NET who underwent PRRT and received SSA as a combination therapy with PRRT and/or as a maintenance therapy showed a significantly higher rate of clinical benefit compared to the PRRT monotherapy [55]. PRRT has also shown durable objective responses in patients with advanced progressive grade 1 or 2 pancreatic neuroendocrine tumours (pNET), who were followed up for a period of 4 years after 4 cycles in a combined ^177^Lu-DOTA-TATE-capecitabine-temozolomide regimen [56]. This confirmed the role of PRRT-chemotherapy combinations as an effective outpatient regimen for control of pNET. PARP inhibitors as potentiators of ^177^Lu-DOTA-TATE have shown huge potential. In human NET cell lines, PARPi potentiates PRRT via augmenting the downstream effect of ^177^Lu-octreotate-induced DNA damage [57]. This was also confirmed in explants of fresh human pancreatic neuroendocrine tissue [58]. As more data emerges, it may soon become common place to administer radionuclide therapy with other systemic therapies such as chemotherapy and immunotherapy.

### 4.3. Combinations with Other Types of Radiation

Several studies have looked at increasing the cytotoxicity of TRT through combination with photon external beam radiation therapy (EBRT) [59,60]. Recently, the impact of scheduling of this type of combination on tumour response has been revisited by Corroyer et al., whose findings emphasized the importance of treatment scheduling according to pathophysiological criteria such as tumour vessel permeability and tumour growth kinetics for TRT-photon EBRT combinations [61]. Melzig et al. found that combining the radiopharmaceuticals ^131^I-Benzamide or ^131^I-cetuximab with high LET carbon ion radiation increased their cytotoxic effects in in vivo models, B16F10 (melanoma) and A431 (skin epidermoid cancer), respectively [62]. The carbon ions activated the immune-response and the DNA damage and cell-cycle control pathways with marked enhancement of the effect of the TRT agents. Combinations of different radiopharmaceuticals may have a positive impact on response to therapy, although the attendant increased radiation exposure carries the risk of normal tissue side-effects. Ahmadzadehfar et al., however, found that administering repeated cycles of ^177^Lu-PSMA-617 for metastatic prostate cancer patients after ^223^Ra-dichloride was safe with only a small probability of heamatotoxicity [63].

## 5. Future Directions

The chemokine receptor subtype-4 (CXCR4) is an interesting new target for TRT, as it is overexpressed in more than 70% of human solid tumours, including breast, prostate, and cervical cancers and B-cell lymphoma, neuroblastoma, melanoma and gliomas. It also represents an additional molecular target for the theranostic approach in patients with SSTR-negative tumours [64]. The first use of cyclic pentapeptide, ^68^Ga-Pentixafor for CXCR4 PET imaging and its therapeutic analog ^177^Lu-Pentixather, in a patient with multiple myeloma showed high tumour-to-kidney and tumour-to-liver uptake ratios of 3.1 and 6.4 [65]. In addition, targeting of the tumour micro-environment is emerging as a promising strategy for overcoming heterogeneous intratumoural radiopharmaceutical distribution. Jin et al. proposed the use of ^64^Cu-RaftRGD which accumulates in regions of angiogenesis and ^64^Cu-ATSM in regions of hypoxic metabolism for glioblastoma [66]. Despite using the same total radioactivity dose of 37 MBq, these authors found the combined use of ^64^Cu-RaftRGD and ^64^Cu-ATSM to cause more efficient inhibition of tumour growth and tumour cell proliferation, and to prolong survival, than either single agent. Targeting efforts against fibroblast activation protein (FAP) present another promising strategy for targeting of the microenvironment of tumours. FAP is expressed in a subpopulation of stromal cells of many solid tumours, and overexpression is associated with poor prognosis in solid tumours [67]. Using radiolabelled FAP-Inhibitors (FAPI) FAPI-02 and FAPI-04, the Haberkorn group demonstrated proof of concept for the clinical use of ^68^Ga-labeled FAPI PET/CT in various cancers with significant lower background uptake in the liver and brain compared to ^18^F-FDG [68,69]. In a proof of principal approach, the therapeutic application of FAP inhibitors was demonstrated in a patient with metastasized breast cancer treated with 2.9 GBq ^90^Y-FAPI-04, which led to a reduction in pain medication at a considerably low dose [70]. FAP is a molecular target that holds great potential for tumour imaging and therapy.

## 6. Conclusions

TRT offers an excellent therapeutic option for many cancer patients. We have paid particular attention in this review to the application of TRT to metastatic NET and prostate cancers, areas that have seen the most promising advances in the last five years. Recent developments have focused on ensuring uptake of a cytotoxic amount of radioactivity into the tumour while minimising the burden on normal healthy tissue through modification of drug specificity and pharmacokinetics. Future avenues of research include incorporation of information about the radiobiological effects of TRT agents into individual dosimetry-based treatment planning.
